# Modeling of a High Force Density Fishbone Shaped Electrostatic Comb Drive Microactuator

**DOI:** 10.1155/2014/912683

**Published:** 2014-07-21

**Authors:** Megat Muhammad Ikhsan Megat Hasnan, Mohd Faizul Mohd Sabri, Suhana Mohd Said, Nik Nazri Nik Ghazali

**Affiliations:** ^1^Department of Electrical Engineering, Faculty of Engineering, University of Malaya, 50603 Kuala Lumpur, Malaysia; ^2^Department of Mechanical Engineering, Faculty of Engineering, University of Malaya, 50603 Kuala Lumpur, Malaysia

## Abstract

This paper presents the design and evaluation of a high force density fishbone shaped electrostatic comb drive actuator. This comb drive actuator has a branched structure similar to a fishbone, which is intended to increase the capacitance of the electrodes and hence increase the electrostatic actuation force. Two-dimensional finite element analysis was used to simulate the motion of the fishbone shaped electrostatic comb drive actuator and compared against the performance of a straight sided electrostatic comb drive actuator. Performances of both designs are evaluated by comparison of displacement and electrostatic force. For both cases, the active area and the minimum gap distance between the two electrodes were constant. An active area of 800 × 300 *μ*m, which contained 16 fingers of fishbone shaped actuators and 40 fingers of straight sided actuators, respectively, was used. Through simulation, improvement of drive force of the fishbone shaped electrostatic comb driver is approximately 485% higher than conventional electrostatic comb driver. These results indicate that the fishbone actuator design provides good potential for applications as high force density electrostatic microactuator in MEMS systems.

## 1. Introduction

Actuators are used to convert nonmechanical input energy into mechanical output energy. Actuators can be used in different scales, ranging from macroscopic actuation through electromagnetic motors, hydraulics and pneumatics, to microscopic actuation where the actuators are of the order of microns for MEMS applications. In MEMS applications, actuators are used to achieve positioning [1], such as positioning a cantilever tip to perform as microgrippers to move miniature objects [2], or to access a specific data point in data storage systems such as in the “Millipede project” [3]. The main parameters that need to be considered for microactuator performance include displacement, response time, load capacity, actuation force, resolution, degrees of freedom, and size [4–7]. The most common actuators are piezoelectric actuators, electromagnetic actuators, electrostatic actuators, thermal actuators, and electrochemical actuators, each with their respective advantages and drawbacks [8–12]. For example, electromagnetic actuators possess a high efficiency in converting electrical energy into mechanical work but are bulky and require a high operating voltage [13]. On the other hand, piezoelectric actuators provide a high actuation force and speed but have low intrinsic displacement [14, 15]. Electrostatic actuators have some favourable performance characteristics, such as a large displacement, as demonstrated by Grade et al. [16] and Liu and Kenny [17]. However, a main drawback of the electrostatic microactuator is its large active area, as a typical electrostatic actuator configuration comprises a large array of interdigitated electrodes which occupy a large surface area.

The mechanism of operation for electrostatic actuators can be described as follows.

A set of interdigitated electrodes (or combs) of opposite polarities are arranged in order to set up an electric field. One set of the combs is fixed, whilst the other set is allowed to move. The resulting electrostatic field produces an electrostatic force to drive the motion of the moveable set of combs. This electrostatic force has components in the *x*-*y* plane (in-plane motion) and the *z*-plane (out-of-plane motion). Typically, the “useful” force is the component along the longitudinal axis of the electrostatic combs, whilst the components of force in the other directions are considered undesirable and lead to side snap-in. However, there have also been novel designs where this “side snap-in” can be utilized for a higher degree of freedom of motion.

The electrostatic actuators are useful for producing a large displacement at low driving voltage [11]. However, its force density only ranges from low to moderate, owing to its large surface area. Strategies to increase the force density include increasing the number of comb fingers, increasing the electrical potential, and reducing the gap between the fixed and moving electrodes. However, each of these strategies has their own drawbacks: increasing number of comb fingers results in an increase in active area, increasing the potential corresponds to an increase in driving voltage, and reducing the gap width has a limitation in terms of fabrication capability [18, 19]. Therefore, it is difficult to fabricate electrodes beyond a minimum size and correspondingly small gap (2-3 *μ*m). In addition, a small gap also results in a higher possibility of lateral instabilities where translational motion, intended to be in, say, the *x*-direction, also results in significant deflection in the *y*-directions and *z*-directions, hence causing the two sets of combs to collide [20].

Therefore, this paper proposes a design which aims to achieve a high displacement and high force density electrostatic comb drive actuator, which is not limited by the issues stated above. This proposed design is fundamentally a branched actuator resembling a fishbone, where a “backbone” has smaller fingers protruding out of it. This design is intended to replace the straight sided comb actuator conventionally used. Introduction of the branched “fishbone” structure increases the cross-sectional area of the electrodes and correspondingly the capacity of the structure. Given that the electrostatic force is the gradient of the electrostatic energy and, in turn, the electrostatic energy is proportional to the capacity under constant voltage conditions, the electrostatic force is inferred to increase with a corresponding increase in capacity. In this paper, the fishbone shape of electrostatic comb drive is proposed to replace the conventional comb driver for high force density performance, through the increase in the cross-sectional area due to the fishbone structure. This paper will first explain the fundamental design of the fishbone actuator, followed by the performance analysis of this design compared to the straight sided actuator.

## 2. The Electrostatic Comb Actuator

**Figure 1:**
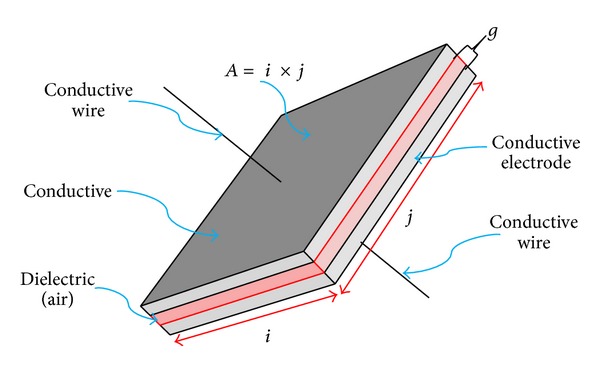
The schematic view of a unit parallel plate actuator (*i* = width, *j* = height).

**Figure 2:**
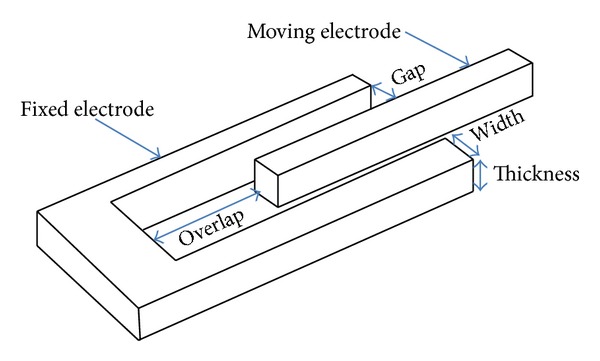
The schematic view of a unit conventional comb drive actuator. All dimensions are represented in *μ*m.

In general, electrostatic actuators can be classified into two types: parallel plate actuators as shown in Figure 1 and electrostatic comb drivers as shown in Figure 2.

A parallel electrostatic actuator is usually made of two electrodes which has two surfaces facing each other. At least one of the two electrodes moves along the direction of the applied electrical field. However, parallel actuators usually have a very limited displacement range. The direction of the displacement along the direction is limited by electrode gap (*g*), defined as the shortest distance between two electrodes. The electrostatic force (**f**) is defined as the force generated in a unit area, *A*, as shown in Figure 1 [21]. Consider

(1) $$\begin{array}{c} f=\frac{\varepsilon V^{2}}{2g^{2}}, \end{array}$$

where **ε** is the dielectric constant, **V** is voltage, and *g* is minimum distance between fixed and moving electrodes.

The electrostatic force generated by a parallel electrostatic actuator is therefore highly nonlinear as it is a function of the electrode gap (*g*). Moreover, a parallel plate actuator usually has a collapse voltage, beyond which the electrodes will collide into each other. This situation limits the displacement to only a portion of the total gap of the actuator, for example, only one-third of the electrode gap (*g*).

Electrostatic comb drivers were designed in order to overcome the nonlinear voltage-displacement characteristics of the parallel electrostatic actuator [22]. A comb electrostatic actuator generates an electrostatic force to drive a movable member of the comb driver. However the existing electrostatic comb drivers have their own challenges. For example, a comb driver usually occupies more space than that of a parallel plate actuator and has a smaller electrostatic force density due to the large active area occupied by the comb structure. The width of comb fingers should be as small as possible to enhance the force density of the comb driver. Comb drive actuators are used to produce large displacement at low driving voltages. The equation governing the drive force generated by an electrostatic comb drive is given as follows [21]:

(2) $$\begin{array}{c} F=\frac{n\varepsilon t}{g}V^{2}, \end{array}$$

where *n* is number of fingers, *ε* is electrical permittivity, *t* is finger thickness, *g* is gap between fingers, and **V** is voltage applied. Note that *n*, *t*, and *g* are the parameters related to the physical dimension of the comb actuator. The objective of the design is to propose an electrostatic actuator which maximizes **F** whilst occupying the least amount of die area. Therefore, *n* and *t* should be as large as possible and *g* should be as small as possible. However, lateral instabilities, where side snap-in results in collision of the two set of combs, become increasingly difficult to overcome as the comb geometry is decreased.

## 3. The Fishbone Shaped Electrostatic Comb Drive Actuator Design

**Figure 3:**
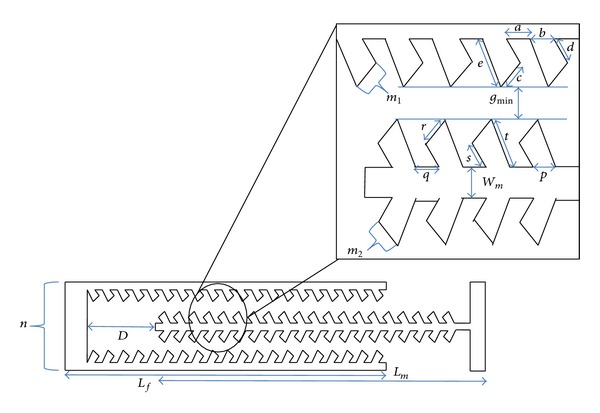
A schematic cross-sectional view of an example of single finger [*n* = 1] of fishbone electrostatic comb driver having 20 sets of small fingers [*m*_1_, *m*_2_ = 20] within and a portion of single unit fishbone electrostatic comb driver.

The design of the fishbone shaped electrostatic actuator is shown in Figure 3. The electrodes are made of silicon. In this figure a single finger acts as a backbone from which a series of smaller fingers protrude, twenty in this particular illustration.

The magnified section of Figure 3 shows the labelling of the respective dimensions for the fishbone structure. The centre electrode is allowed to be movable, whilst the upper and lower electrodes are fixed. Angles **α**_1_, **α**_2_, and **α**_3_ define the angle of the protruding small fingers of the fixed electrode, which can range from 0 to *π*. Similarly, angles **β**_1_, **β**_2_, and **β**_3_ define the angles for the protruding small fingers of the moving electrode, which again can range from 0 to *π*. *g*_min⁡_ represents the minimum perpendicular distance between the fixed and moving electrodes. The width of the central backbone of the fixed electrode is defined as *w*_*f*_.  Table 1 shows the details of the design.

**Figure 4:**
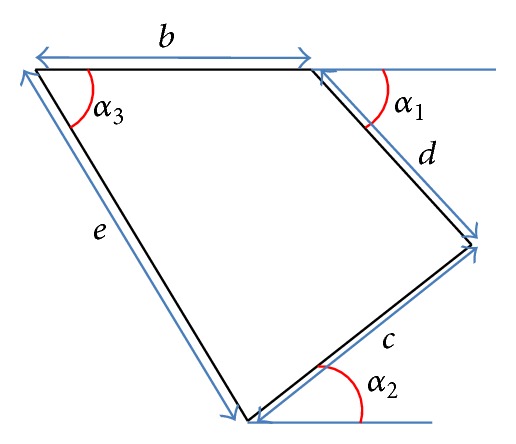
Example of small fingers which are placed at angle **α**_2_ connected to the electrode.

For practical purposes, that is, realistic fabrication of this microactuator design based on conventional MEMS processes, a minimum dimension for the protruding finger (*w*_*m*_ and *w*_*f*_) of five *μ*m was defined. The choice of angles for **α**_1_, **α**_2_, and **α**_3_ and **β**_1_, **β**_2_, and **β**_3_ was determined by taking into consideration this minimum dimension. For example, referring to Figure 4, using a minimum parameter of 5 *μ*m (for *w*_*m*_ and *w*_*f*_), the possible choices of angles were 60°, 30°, and 65°.

The displacement of the actuator is limited by the length of the fixed electrode, as the two sets of electrodes will collide beyond this value. The two designs compared in this paper are shown in Figures 2 and 3, for the straight sided and fishbone actuators, respectively. For the straight sided design, the actuator dimensions are as follows: the length *L* is 200 *μ*m, the width *W* is 5 *μ*m, the finger length is 205 *μ*m, the finger width is 5 *μ*m, the overlap length is 100 *μ*m, the gap is 5 *μ*m, and the thickness is 100 *μ*m.

Referring to Figure 3, the fishbone shaped electrostatic comb drive actuator is a modification from conventional comb drive actuator shown in Figure 2 by maintaining similar dimensions for the gap, width *W*, length *L*, finger length, finger width, thickness, and the overlap length but covering a higher cross-sectional area. The overall comb design achieves a fishbone look, where small fingers are attached at an angle to the backbone electrode, for both the fixed and the moving electrodes.

## 4. Performance Comparison of Multiple Fingers with Similar Cross-Sectional Area

Two electrostatic drive microactuator configurations are considered for comparison: one containing 16 fingers of the fishbone shaped comb driver and the other containing 40 fingers of conventional comb driver, both of an almost similar cross-sectional area of approximately 800 × 300 *μ*m. The main objective of this simulation is to deduce the force density and displacement of the two kinds of electrostatic comb actuators, for a similar active area. The gap spacing (5 *μ*m) and overlap (100 *μ*m) are kept constant for both cases. Both microactuator configurations were modelled for the electromechanical motion through finite element simulation using Comsol Multiphysics 4.3. Driving voltage was applied to the moving electrodes, ranging from 0 V to 100 V and the fixed electrodes were grounded. The output of this simulation is the drive force produced by the electrostatic coupling of the electrodes.

## 5. Spring Design

**Figure 5:**
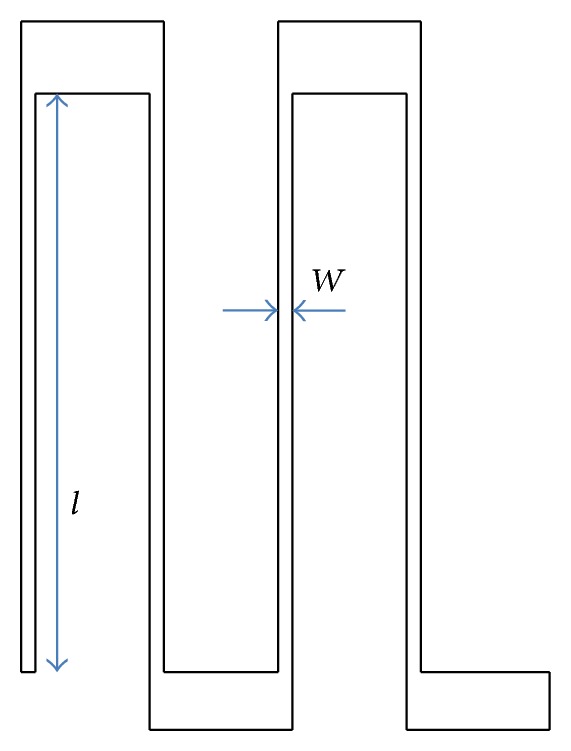
A close-up of the serpentine spring used in the simulation.

A spring component was built into the actuator design to provide restraining force and flexibility to the actuator motion. The serpentine spring type was selected for both designs of comb drive actuators for actuation and deformations studies, as it in particular provides good restraining force against the electrostatic force acting in the* y*-direction, which is a main cause of instability. The close-up of this serpentine spring can be seen in Figure 5.

This type of spring can provide good linear behaviour of displacement for the desired *x*-direction and simultaneously provide stiffness for undesired translation in the *y*-direction. **K** is the mechanical stiffness of the spring, which is defined by [23]

(3) $$\begin{array}{c} K=\frac{Et}{N}{\left(\frac{w}{l}\right)}^{3}, \end{array}$$

(4) $$\begin{array}{c} F=K\Delta x, \end{array}$$

where *E* is young modulus, *t* is thickness of the spring, *N* is the number of cantilever beams of the spring, *w* is width of the beam, *l* is the length of the spring, **F** is mechanical force, and Δ*x* is spring displacement. All the parameters of the spring below were used in (3) and the theoretical value for a serpentine spring, using parameters defined in Table 2, is 0.034 N/m.

**Figure 6:**
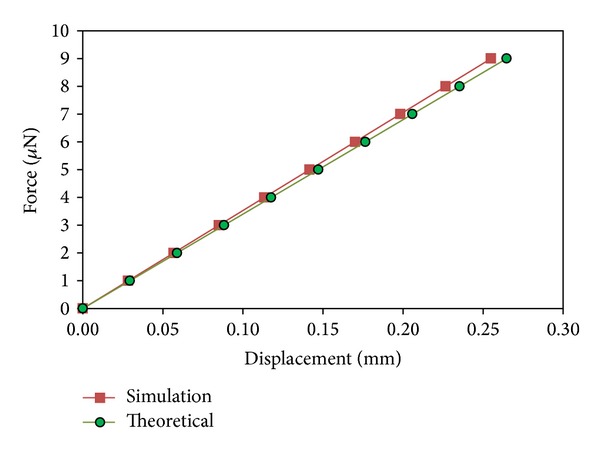
Simulation result of spring displacement with force applied.

The simulation results are shown in Figure 6, against the theoretical calculations, which show good agreement. The simulation results indicate that the stiffness of the serpentine spring is 0.035 N/m, which correspond to a small 2.9% difference between the theoretical and simulation values for spring stiffness, which is acceptable. This difference is believed to be due to the residual inaccuracy of the meshing.

## 6. Finite Element Simulation of Electrostatic Comb Actuator

### 6.1. Governing Equations

In order to evaluate the performance of the actuator, finite element analysis was conducted using COMSOL Multiphysics. The objectives of this simulation are to predict the actuator displacement and force for both the conventional straight sided electrostatic comb actuator and the fishbone shaped actuator in this design. First, simulation of a single finger of the straight sided actuator and fishbone shaped actuator was performed, and their performances were compared. Next, simulation of an array of straight sided and fishbone shaped actuators was performed, for a fixed active area for the two cases in order to compare the performance of the two types of electrostatic comb actuators.

The simulation was chosen to be in 2D as the main investigation for this paper is the translational force and displacement in the *XY* plane. The model applies an electric potential for the moving electrode and grounds the fixed electrode.

The electrostatic comb driver is capacitive device where the electrostatic problem at hand is represented by Laplace's equations to find potential distribution (**V**) of the geometry [7]. Consider the following:

(5) $$\begin{array}{c} \nabla^{2}V=\frac{{\partial V}^{2}}{{\partial X}^{2}}+\frac{{\partial V}^{2}}{{\partial Y}^{2}}=0. \end{array}$$

The electric energy **W**_**e**_ and capacitance **C** are solved for all element nodes, based on

(6) $$\begin{array}{c} W_{e}=\frac{1}{2}\varepsilon_{0}\iint {\left|E\right|}^{2}dX\;dY,\\ C_{t}=\frac{2}{V^{2}}\iint W_{e}dX\;dY, \end{array}$$

where **C**_**t**_ is the capacitance per unit thickness.

The electrostatic force between comb fingers is related to the voltage and capacitance changes, as the physical motion of the actuator continuously induces a change in the spacing and overlap, which has a direct impact on the values of capacitance and voltage. The relationship between electrostatic force **F**_es_ and displacement is related to the rate of change of the electrostatic energy in the direction of motion (*x*) and consequently related to the rate of change of capacitance in the *x*-direction (for a fixed voltage) as follows:

(7) $$\begin{array}{c} {\;F}_{\text{es}}=-\frac{\partial W_{e}}{\partial x}=\frac{\partial C\left(x\right)}{\partial x}\cdot \frac{V^{2}}{2}, \end{array}$$

where **W**_**e**_ is electric energy and **F**_es_ is the electrostatic force.

### 6.2. Meshing

The materials selected were silicon for the electrodes and air for the surrounding areas. The boundary conditions were selected to be fixed to ground for the static electrodes and the electric potential, ranging from 0 to 100 V for the moving electrode. The external boundaries surrounding the conductor are selected to be air and to contain zero charge, as represented by the following equation:

(8) $$\begin{array}{c} -\overset{⃑}{n}\cdot D=0, \end{array}$$

where $\overset{⃑}{n}$ is the unit normal vector and **D** is electric displacement.

**Figure 7:**
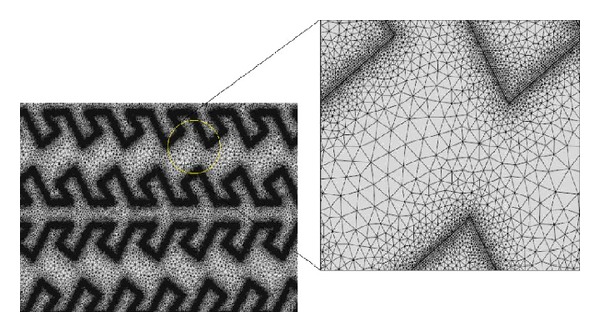
Schematic of mesh generated after extra refinement is made at the edge surface of the fishbone shaped electrostatic comb driver fingers.

Meshing was then performed on the actuator geometry. Based on the work by Harouche and Shafai [18] for an electrostatic comb actuator system, a mesh of free triangular mesh with quadratic elements which consist of 1676886 numbers of elements was chosen to ensure convergence. For the fishbone shaped electrostatic comb drive actuator design, which contains edges compared to the conventional straight comb finger of comb driver, extra refinement of element size was made in the critical regions as shown in Figure 7.

### 6.3. Module Selection for Simulation

First, the electrostatic force in the actuator system was simulated using the “Electrostatic Analysis in AC/DC module.” Second, once the electrostatic force due to a driving voltage was set up for the electrostatic actuator, the value of this force was coupled to the “Solid Mechanics” analysis under the Structural Mechanical Module. This second step drives the actuator motion. For this step, the incorporation of the spring discussed in Section 5 is essential to ensure a restraining force to the actuator motion. Third, the displacement due to this actuator motion is obtained through adjustment of the actuator system geometry through the use of a “Moving Mesh” model.

## 7. Results and Discussion

### 7.1. Comparison of Driving Force and Displacement for a Single Straight Sided and Fishbone Electrostatic Comb Actuator

**Figure 8:**
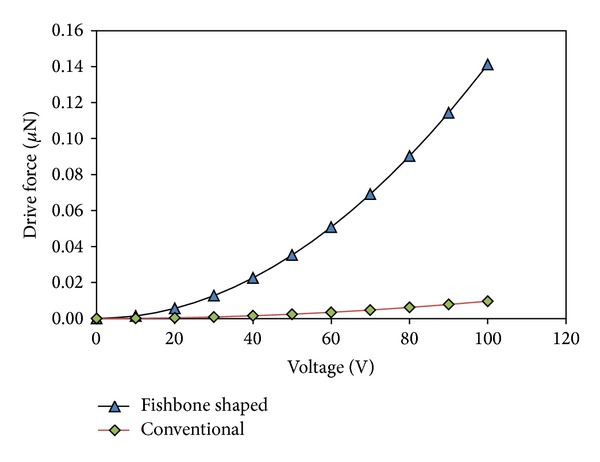
Electrostatic force (drive force) as a function of voltage, exerted on the comb fingers in the *x*-direction for both single unit of straight sided comb finger and fishbone shaped comb driver.

**Figure 9:**
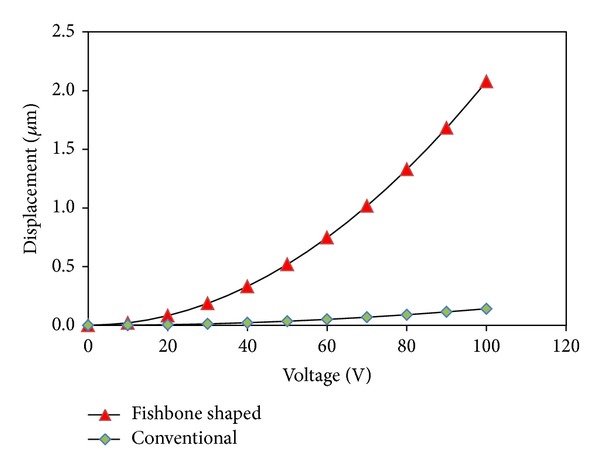
Driving voltage versus displacement of the comb fingers in the *x-*direction for single unit of straight conventional comb finger and fishbone shaped comb driver.

First, the performance of a single straight sided and fishbone actuator is compared in terms of its driving force and displacement upon application of an external voltage. For this simulation, an external voltage ranging from 0 V to 100 V was applied.  Figure 8 shows the quadratic relationship between the electrostatic force acting vertically on a single straight sided comb finger and fishbone shaped comb driver, respectively, when external voltage is applied. For example, at 100 V driving external voltage, the drive force for fishbone shaped comb driver is 1364% higher than the straight sided comb driver for a single finger. Similarly, Figure 9 shows a comparison of the relationship between the electrostatic force and displacement, for a single straight sided comb finger and fishbone shaped comb finger. For 100 V, the improvement in displacement using the fishbone comb finger was 1364%.

**Figure 10:**
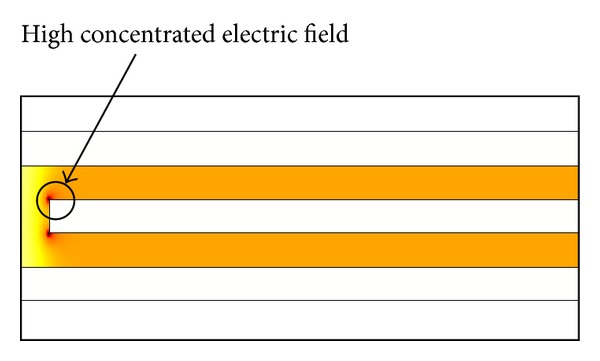
Electric field distribution of single finger straight electrostatic conventional comb drive actuator.

**Figure 11:**
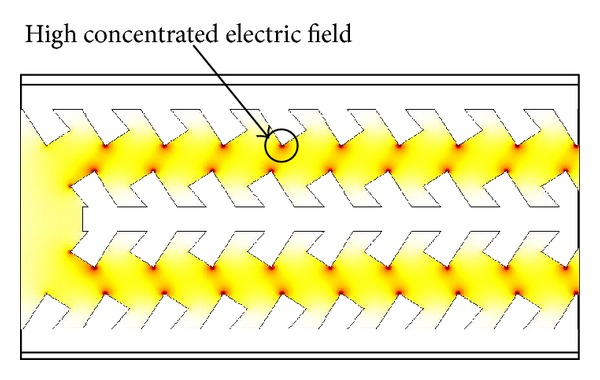
Electric field distribution of single finger fishbone shaped electrostatic comb drive actuator.

The improvement in displacement can be directly attributed to an understanding of the electric field distribution of the comb actuator geometry. The electric field distribution of the straight sided and fishbone actuators is shown in Figures 10 and 11, respectively, in order to highlight the differences in the electrostatic field for both designs. In the case of the straight sided actuator shown in Figure 10 it can be seen that the electric field is concentrated at the corners of the straight comb finger. The magnitudes of the electrostatic forces on the static and dynamic electrodes are equal but in the opposite directions, this cancels out the electrostatic forces in the *y*-direction. The only remaining forces are at the corners of the fingers, in which the largest forces or more are commonly known as fringing forces that ultimately serve as the main driving force of the straight sided electrostatic comb driver. In comparison, the fishbone shaped comb drive actuator shown in Figure 10 produces many more strong electrostatic fringing forces, due to the many sharp edges of the actuator geometry. These highly concentrated electric field regions can be referred to in Figure 11.

Furthermore, since these small fingers are placed at an angle to the backbone, the resultant electrostatic fields have components in both *x*- and *y*-directions. Whilst the forces in the *y*-directions will cancel out similar to the case of the straight sided comb actuator, the components in the *x*-directions are added up to form strong driving force in the *x*-direction. This increase in forces due to the fishbone geometry is responsible for the significant increase in actuation force and displacement for the fishbone geometry.

### 7.2. Comparison of Driving Force and Displacement for a Single Straight Sided and Fishbone Electrostatic Comb Actuator

**Figure 12:**
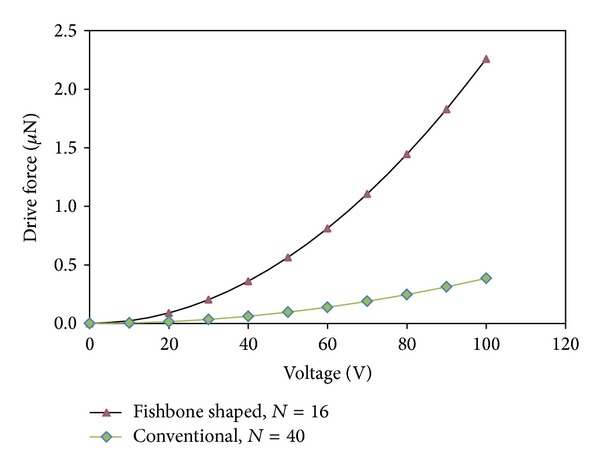
The relationship between driving voltage and electrostatic force exerted on the comb fingers in *x*-direction for both multiple fingers of straight conventional comb driver and fishbone shaped comb driver.

**Figure 13:**
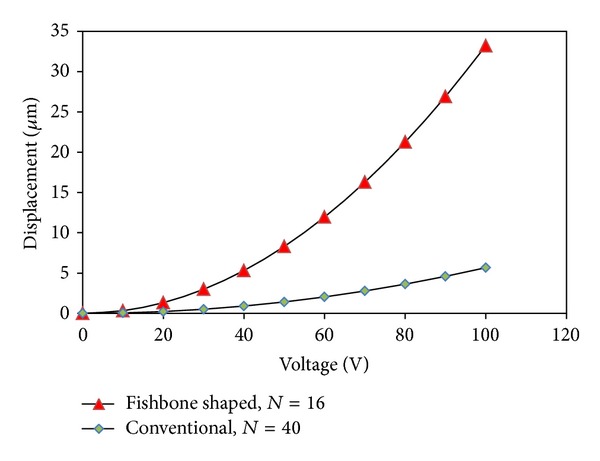
The relationships between driving voltage and displacement of the comb fingers in *x-*direction for both multiple fingers of straight conventional comb driver and fishbone shaped comb driver.

The performance of an array of straight sided and fishbone actuators, contained with an equivalent active area (of 800 × 300 *μ*m), is compared in terms of its driving force and displacement upon application of an external voltage. For this simulation, an external voltage ranging from 0 to 100 volts was applied.  Figure 12 shows the relationship between the electrostatic force acting vertically on a single straight sided comb finger and fishbone shaped comb driver, respectively, when external voltage is applied. For example, at 100 V driving external voltage, the drive force for fishbone shaped comb driver is 485% higher than the straight sided comb driver for a single finger. Similarly, Figure 13 shows a comparison of the relationship between the electrostatic force and displacement, for a single straight sided comb finger and fishbone shaped comb finger. For 100 V, the improvement in displacement using the fishbone comb finger was 485%, due to the use of the same spring constant.

**Figure 14:**
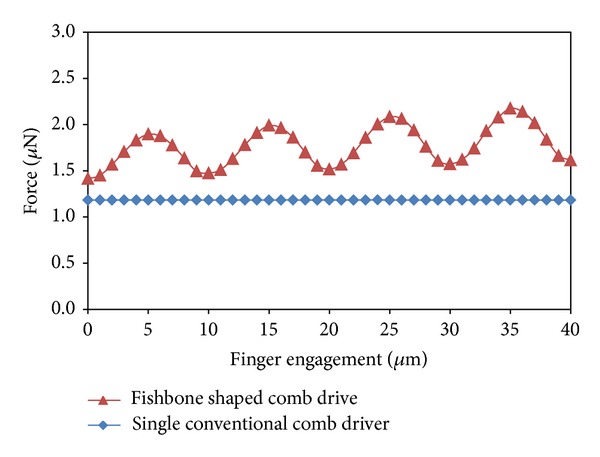
Comparison of force versus finger engagements for conventional comb driver and fishbone shaped comb actuator.

Figure 14 shows the periodic oscillation of the electrostatic force as a function of finger engagement, where finger engagement is defined as the distance *D* shown in Figure 3 previously. For a single finger, the oscillation may be in the range of 0.5 *μ*N, which is equal to 13% of the average electrostatic force. It is significant to note that the characteristic behaviour of the electrostatic force of this fishbone actuator, as a function of displacement, results in a periodic variation with regard to distance. We have identified this as a cogging effect, as shown in Figure 14. This cogging effect may be linearized or manipulated to other force-displacement profiles (such as a stepped profile) through manipulation of the electrode arrays.

## 8. Conclusion

This work discusses the comparison of a fishbone shaped electrostatic actuator and compares its performance with a straight sided electrostatic actuator. Comparison in terms of driving force and displacement as a function of voltage was presented giving an improvement 1364% for a single actuator operating at 100 V and 485% improvement on the performance for a similar active area of electrostatic actuator. This indicates that this fishbone design has potential as a high density electrostatic actuator, which is a solution to the high active area which challenges most designs related to electrostatic actuator. This high force density is enabled through the branched structure of fishbone actuator geometry, which increases the driving force along the longitudinal axis of the actuator and at the same time minimizes any crosstalk in the transverse (*y*) direction. Such a high force density microactuator has potential for use in MEMS applications such as microgrippers and micropositioning systems.

## Figures and Tables

**Table 1:** Parameters of basic fishbone shaped comb drive.

Parameters	Value
Angles **α**_1_, **α**_2_, and **α**_3_	60°, 30°, and 65°
Angles **β**_1_, **β**_2_, and **β**_3_	60°, 30°, and 65°
Minimum gap, *g*_min⁡_	5 *μ*m
Horizontal displacement, *D*	100 *μ*m
Fixed electrode width, *w*_*f*_	5 *μ*m
Moving electrode width, *w*_*m*_	5 *μ*m
Number of small fixed fingers, *m*_1_	20
Number of small moving fingers, *m*_2_	20
Length of moving electrode, *L*_*m*_	205 *μ*m
Length of fixed electrode, *L*_*f*_	205 *μ*m
Length of *a*, *b*, *c*, *d*, *p*, *q*, *r*, and *s*	5 *μ*m
Length of *e* and *t*	8 *μ*m

**Table 2:** Parameters of spring design.

Parameters	Values
Young's modulus, *E*	1.70 × 10^11^ Pa
Thickness, *t*	100 *μ*m
Number of cantilever beams, *N*	4
Width, *w*	2 *μ*m
Length, *l*	1000 *μ*m
